# Asymptomatic* Plasmodium* Parasites among Adults in Eastern Uganda: A Case of Donor Blood Screening at Mbale Regional Blood Bank

**DOI:** 10.1155/2018/6359079

**Published:** 2018-07-09

**Authors:** Simon Peter Inyimai, Mosses Ocan, Benjamin Wabwire, Peter Olupot-Olupot

**Affiliations:** ^1^Mbale Regional Blood Bank, Uganda; ^2^Mbarara University of Science and Technology, Uganda; ^3^Mbale Clinical Research Institute, P.O. Box 1966, Mbale, Uganda; ^4^Busitema University, Faculty of Health Sciences, Mbale Campus, P.O. Box 1460, Mbale, Uganda

## Abstract

**Background:**

There is a paucity of data on asymptomatic carriage of* Plasmodium* parasite among adult population in Eastern Uganda, an area of perennial high transmission of malaria. In this study, we estimated the prevalence of* Plasmodium* parasites in donor blood units at Mbale Regional Blood Bank (Mbale RBB), a satellite centre of the Uganda Blood Transfusion Service (UBTS).

**Method:**

This was a cross-sectional descriptive study in which 380 screened donor blood units were examined for the presence of* Plasmodium* parasites. A systematic random sampling technique using the interval of 7 was used in selecting the screened blood units for testing. Two experienced malaria slide microscopists (MC1 and MC2) independently examined each thick and thin blood slide under high power magnification of X400 and then X1000 as stated on the study standard operation procedure (SOP). Each slide was examined for 100 oil immersion fields before the examiner declared them negative for* Plasmodium* parasites. The results by each microscopist's examination were tallied separately, and finally, the two tallies were compared. The third independent microscopist (MC3) was blinded to the results from MC1 and MC2, but whose role was to perform quality control on the slides randomly sampled and read 38 (10%) of all the slides and was available to examine any slides with inconsistent findings by MC1 or MC2.

**Results:**

All the microscopists were unanimous in all the slide readings. Five of the thick smears (1.3%) confirmed the presence of* Plasmodium* parasites among donor blood units. Of these, 4/5 were from male donors.* Plasmodium falciparum* was identified in 4 positive samples, while* Plasmodium malariae* was identified in one of the donor units.

**Conclusion:**

The 1.3% prevalence of* Plasmodium malaria* parasites in screened donor blood units represents risk of malaria blood transfusion transmitted infection and a pool of community transmittable malaria infections, respectively.

## 1. Background

Globally, there has been a substantial reduction in malaria incidence as a result of scaling up of control and prevention efforts. There are currently renewed interests on initiatives for malaria elimination [1, 2] and accurate mapping of areas with malaria parasite prevalence [3]. This is especially in this era of malaria epidemiological transition in which some countries in Africa have documented either no decline [4], an increase in hospitalisations with severe* Plasmodium falciparum* (*P. falciparum*) malaria during the same period [5], or a resurgence of severe malaria following a period of sustained control [6]. Worldwide* P. falciparum* malaria causes 300–500 million clinical episodes, with up to 445,000 direct deaths attributed to the disease in 2017 [7]. In addition, malaria is a major coinfection to other diseases contributing to an estimated 3 million deaths annually across the world [8]. Ninety percent of these deaths occur in African children <5 years old [9, 10]. Fifteen sub-Sahara African (SSA) countries account for 80% of the global malaria cases [7].

Control and/or prevention of malaria using vaccines has so far proven to offer limited short-term protection [7]. Thus, single-phased prevention strategies alone in many parts of Africa will not adequately contribute to substantial reduction in the disease burden. Strategies to limit malaria transmission in the community by breaking the malaria cycle and disrupting any further transmission have provided promising outcomes in malaria control and elimination efforts in malaria endemic areas [11]. However, these efforts require a combination of approaches and not a single intervention at a time [12]. Use of the once effective indoor residual spraying with Dichlorodiphenyltrichloroethane (DDT) in some endemic countries has been discouraged because of its potential health and environmental hazards [13], while in other settings, growing resistance to DDT has been reported [12]. Malaria control and elimination efforts need to be expanded to other previously recognised but poorly described risk of malaria transmission in communities, for instance, transmission of malaria through blood transfusion, especially in malaria endemic regions of the sub-Saharan Africa [3]. Targeting malaria transfusion transmitted infections will enhance the gains being reported from the current malaria control and elimination efforts [7]. Therefore, understanding the prevalence of both symptomatic and asymptomatic malaria would help inform targets for control and prevention efforts geared towards elimination of the disease. In Eastern Uganda available data demonstrates that spleen rates among children <5 years were high [14], and malaria transmission is intense [14, 15]. There are no data on asymptomatic malaria carriage in adults in Eastern Uganda, just as in the rest of the country. Therefore, descriptions of human populations that harbour malaria parasites are critical. There are some studies evaluating use of chemotherapy for community control of malaria [16]. Other interventions for possible eradication of malaria parasitaemia in the community are being evaluated. The effectiveness of such interventions would be well informed by the understanding of the pools of asymptomatic carriage of malaria parasites in the community. Robust surveillance for asymptomatic carriage of malaria parasites, however, is largely lacking. Exploration of proxy estimation methods could yield useful data for mapping communities at risk or those that harbour potentially transmissible* Plasmodia*. We explored the potential of using Mbale RBB in Mbale municipality with a catchment of 27 districts in the region, as a hub for surveillance for asymptomatic carriage of* Plasmodia*. The facility receives blood units from two categories of voluntary donors; the “walk-in-donors” who voluntarily come to the regional facility to donate, and “out-reach-donors” who are accessed by the Mbale RBB mobile blood collection teams for blood donation in the communities in the catchment area including students. The blood collection teams are trained in counseling and selection of low risk blood donors in the community. Only healthy asymptomatic donors are recruited and these therefore comprise the right target population for surveillance for asymptomatic* Plasmodia* carriage. Before blood is considered safe and issued for transfusion, tests for blood group, viruses (HIV, Hepatitis B, and Hepatitis C), and syphilis (TPHA) are done. We have previously used data on viral screening from Mbale RBB to describe epidemiology of Hepatitis B and Hepatitis C in the communities [17]. Tests for haemoparasites such as* Plasmodium* are not routinely done; therefore, there is no data to describe asymptomatic malaria using donor blood in Eastern Uganda. This study was therefore designed to investigate the feasibility of surveillance for asymptomatic malaria in donor blood as proxy to estimation of community asymptomatic carriage of malaria in a malaria perennial high transmission Eastern Uganda.

## 2. Materials and Methods

This was a cross-sectional study conducted from 1 June to 31 July 2015 in Eastern Uganda. Screened donor blood units from Mbale RBB were examined for the presence of malaria parasites. The region has a stable high transmission burden for malaria with over 100 infective mosquito bites per person per year [15]. The Mbale RBB handles more than 30000 voluntary blood donations per annum.

### 2.1. Sample Size Calculation

Using the Kish, L. (1965) Survey Sampling; John Wiley and Sons, Inc. New York formulae, a sample size of 380-donor blood units was included in the study. The blood donor units that met the selection criteria were serially arranged. The blood units selected for testing were determined by referring to the Mbale RBB database. The database showed that out of the 30000 collected each year, 90% (27000) of them would be safe for blood transfusion.

### 2.2. Sampling Criteria

The study was conducted over 2 months for which a conservative blood collection forecast (monthly minimum 1250–maximum 2750 blood units) was made arriving at a study population of 2530 blood units. The study population of 2530 blood units was divided by the sample size of 380, obtaining a sampling interval of 7. The first unit to be selected was determined by putting pieces of paper containing numbers of the first ten units in a box. One paper piece was then picked at random and the number contained therein was considered as the first number. Every seventh unit, beginning from the currently selected blood unit number, was considered for the assay. Blood samples were extracted from the integral segment of the donor bag for analysis. All blood donor units that showed any form of hemolysis were excluded.

### 2.3. Data Collection

The demographic information of the blood donors without their personal identification information was collected using a data abstraction tool. From each selected blood donor unit, at the integral tubing, about 100*μ*l of blood was collected into a cryovial with the help of a tube sealer. Thick and thin blood smears were made for each blood unit for identification and typing of* Plasmodia* parasites, respectively. The thick blood smears were stained with 10% Giemsa at PH 7.2, a stable methanol based Romanowsky stain. The stained blood smears were examined microscopically under X400 and X1000 objectives. The presence of* Plasmodia* parasites was noted based on their staining features with Giemsa stain. We further quantified the malaria parasites by examining microscopically the X100 objective. The number of* Plasmodium* parasites in the positive smears was counted against 500 WBC and the value that was obtained was multiplied by 16 in order to obtain the* Plasmodium* parasitaemia level per microlitre of blood. We identified the* Plasmodia* species using thin smears. The positive smears were given arbitrary alphabetical letters A, B, C D, and E for confidentiality. Thin blood smears were stained with 10% Giemsa at PH 7.2, a stable methanol based Romanowsky stain and examined as described above.

### 2.4. Quality Control

The quality of results was ensured by staining freshly prepared Giemsa stain. The standard operating procedures of staining were observed in order to obtain consistent quality results. Two laboratory technologists read each pair of thin and thick slide preparation independently. The study SOP provided for a third independent microscopist whose duty was to ensure quality control by randomly sampling and reading 38 (10%) of all the slides and to examine any slides with inconsistent findings by any of the first two microscopists.

### 2.5. Data Management and Analysis

The collected data was entered into Excel spread sheet 2007. Data was then transferred to SPSS* ver* 20.0 for analysis. Proportions were generated from the datasets.

### 2.6. Ethical Approval

The study was reviewed and approved by the Mbarara University of Science and Technology, Faculty of Medicine Research Ethics Committee (FRC), permission to conduct the study was granted by the Principal Medical Officer of Mbale RBB, and further ethical review and approval was done by the Mbale Regional Referral Hospital Research and Ethics Committee (MRRH-REC).

## 3. Results

**Figure 1:**
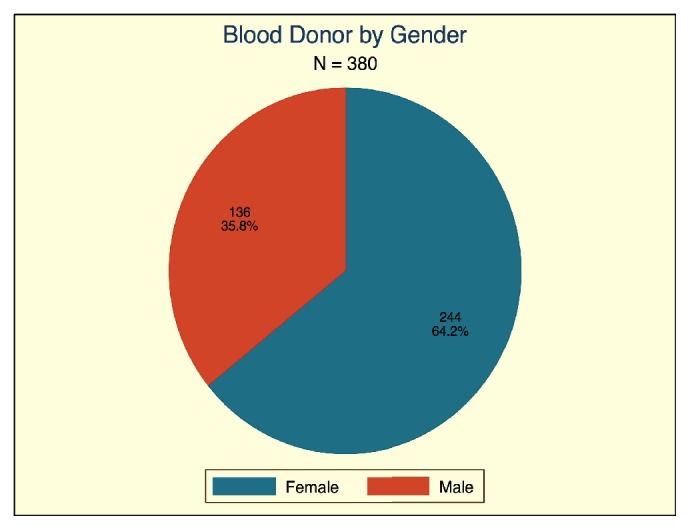
A pie chart of blood donors by gender.

Three hundred and eighty thick and thin blood films were made. A majority of the blood donors were males (Figure 1).

The mean age of the blood donors was 21 ± 4 years, range 18 – 22 years (Table 1).

### 3.1. The Distribution of Samples from Each District in the Catchment Area

**Figure 2:**
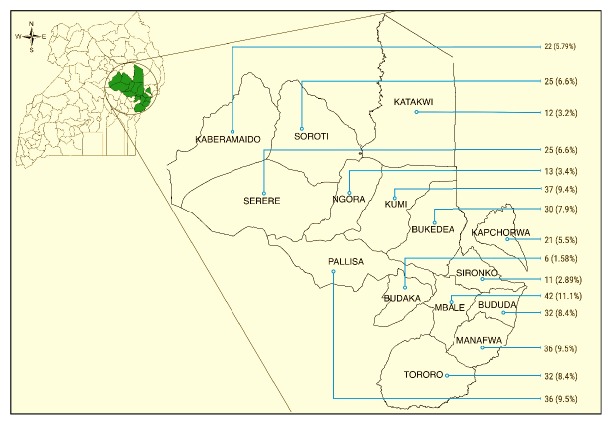
Infographic map of Uganda with insert showing the distribution of blood units sampled for the study.

The donor blood units sampled were from more than half (out of the total 27 districts) of the districts that are covered by Mbale RBB collection teams. Generally, there was relative uniformity in the number of blood units. Nonetheless, a slightly larger proportion of the blood units were collected from donors within Mbale district; 42 (11.1%) units were followed by Kumi district with 37 (9.74%) units. The rest of the distribution is reflected in Figure 2.

The state of* Plasmodium* parasite parasitaemia among the blood units studied was summarized in Table 2.

### 3.2. Prevalence of* Plasmodium* Parasites in the Donor Blood Units

Of the 380 donor blood units screened, 5/380 (1.3%) carried Plasmodium parasites. Of the 5 donor blood units that had* Plasmodium* parasites, a majority (4/5) had* Plasmodium* falciparum, while only one had* Plasmodium malariae* parasites. Of the 136 donor blood units from female donors, 01 (0.79%) had* Plasmodium* parasites while of the 244-donor blood units from male donors 04 (1.6%) had* Plasmodia* parasites (Table 2).

## 4. Discussion

Our results indicate that the prevalence of* Plasmodium* parasites among ready-to-transfuse blood units was 1.3%. This was lower than expected in this malaria high transmission region. This is epidemiologically and immunologically plausible, especially given the fact that individuals who had potentially acquired immunity to the disease may have been sampled. Furthermore, the screening criteria used to identify potential blood donors may have been very stringent that it eliminated a large proportion of asymptomatic carriers in the community. Nonetheless, the 1.3% represents a malaria pool with double the chance of transmitting the disease at the time of donation in the community, that is, through mosquito bites, and in the hospital through blood transfusion. In comparison, our findings are far below the immunological assays for similar surveys. For instance, the malaria antibody prevalence of 7.6% in Saudi Arabia suggests a possible lower diagnostic accuracy of blood slide for malaria compared to immunological assays, lower endemicity notwithstanding. But in West Africa, in malaria endemic setting, malaria in donor blood was 7% [18] and 21% [19], despite similarity in age of donors at these and out sites. This could be due to the differences in blood donor screening practices on one hand but conversely may be due to the differences in malaria seasons or immunity in the donors.

As expected for this region, the dominant species was* P. falciparum* malaria with 4/5 positive samples confirming positivity to the speciation. This is consistent with other series in Africa [20]. The role of asymptomatic parasitaemia in the transmission of malaria and whether or not it requires treatment remains poorly understood. We found low parasitaemia, probably noninfectious [21], but how long these parasites survive in immune human beings, reproduce, and eventually get to transmittable threat holds requires additional studies. Our thinking is that, all asymptomatic parasitaemia is a potential source of infection in the community.

In our study we also found* P. malariae* a rare malaria parasite in Uganda. Furthermore, because of its long incubation period and less severe clinical features, it is likely that the carriers of* P. malariae* infection do not show up for medication early, or if they do they do not receive appropriate medication for this parasite. Consequently, it continues to multiply in the body with a potential, overtime, to cause significant prevalence and/or coinfections with* falciparum*. We found that blood group O accounted for 4/5 cases of asymptomatic malaria (Table 2). This is consistent with another series that indicated a strong association between blood ABO group with asymptomatic malaria* P=*0.022 and a high rate of parasitaemia in blood group O;* P=*0.003 [22]. In our study, we did not look at other factors, but elsewhere, in similar malaria transmission settings, thrombocytopaenia (<150 X10^9^) was associated with asymptomatic malaria among blood donors [19]. The implication of our results with regard to blood transfusion and malaria control is potentially significant because asymptomatic malaria carries the risk of transmitting malaria parasites to the recipients of these blood units, of whom the majority are pregnant mothers and immunologically naive children. Our observations have demonstrated* Plasmodia* in screened donor blood. We recommend better and robust ways of screening for malaria in donor blood to protect blood recipients from these infections [23]. Programmes that target asymptomatic malaria pool should be included in community malaria control programs.

## 5. Conclusion

Malaria causing parasites were present in 1.3% of the donor blood units. This is of clinical and public health significance since it represents a risk of community transmission of malaria from blood transfusion, and also through mosquito bites. Use of more efficient, highly sensitive, and specific diagnostics techniques would improve the accuracy of asymptomatic malaria surveillance using donor blood.

## Figures and Tables

**Table 1:** The age ranges of the blood donors.

**Age Group**	**Number (%)**
18-22	282 (74.2)
23-27	66 (17.4)
28-32	22 (5.8)
33-37	07 (1.8)
38-42	02 (0.5)
43-47	01 (0.3)
Total	380 (100.0)

**Table 2:** Blood group, malaria infection and asymptomatic parasite count.

**Case **	**Age group**	**Gender**	**Blood group**	**Number of Plasmodium parasites/** ***μ*** **l of blood**	**Plasmodium species**
**Case ** **1**	18-23	MALE	O+	48	*P. falciparum*
**Case ** **2**	18-23	FEMALE	O+	62	*P. falciparum*
**Case ** **3**	24-30	MALE	O+	48	*P. malariae*
**Case ** **4**	18-23	MALE	O-	80	*P. falciparum*
**Case ** **5**	18-23	MALE	AB+	32	*P. falciparum*

## Data Availability

The data used to support the findings of this study are available from the corresponding author upon request.
